# Comorbidity and Procedural Factors Associated With Auditory Brainstem Response Outcomes in Children Under Anesthesia

**DOI:** 10.7759/cureus.101697

**Published:** 2026-01-16

**Authors:** Christina Zhu, Henna Tiwary, Ashwini M Tilak, Sophia I Jaguan, Hengameh Behzadpour, Susan Verghese, Tracey Ambrose, Diego Preciado, Brian K Reilly

**Affiliations:** 1 Otolaryngology - Head and Neck Surgery, Georgetown University School of Medicine, Washington, D.C., USA; 2 Otolaryngology - Head and Neck Surgery, George Washington University School of Medicine and Health Sciences, Washington, D.C., USA; 3 Otolaryngology - Head and Neck Surgery, Children's National Hospital, Washington, D.C., USA; 4 Otolaryngology - Head and Neck Surgery, University of Miami School of Medicine, Miami, USA; 5 Anesthesiology, Children's National Hospital, Washington, D.C., USA; 6 Hearing and Speech, Children's National Hospital, Washington, D.C., USA; 7 Otolaryngology, Children's National Hospital, Washington, D.C., USA

**Keywords:** auditory brainstem responses, medical comorbidities, pediatric anesthesia, pediatric hearing loss, sevoflurane vs propofol

## Abstract

Introduction

We aim to evaluate the influence of patient comorbidities and procedural context (standalone vs. combined procedures) on auditory brainstem response (ABR) outcomes in children and to explore whether anesthesia type (propofol vs. sevoflurane) contributes to differences in hearing thresholds.

Methods

A retrospective chart review was conducted of 403 pediatric patients who underwent sedated ABR testing between October 2020 and 2023 at a tertiary children’s hospital. Demographic, anesthetic, and clinical variables, including neurological, cardiac, and respiratory comorbidities, were analyzed. Hearing outcomes were categorized as normal versus any hearing loss across click and tone-burst frequencies. Logistic regression analyses examined associations between hearing loss, anesthesia type, comorbidities, and procedural context, adjusting for American Society of Anesthesiologists (ASA) classification.

Results

No significant association was found between anesthesia type and ABR thresholds after adjusting for ASA class. In contrast, children with cardiac (36% vs. 53%; p=0.008) or respiratory (40% vs. 57%; p=0.013) comorbidities were significantly more likely to exhibit hearing loss. Patients undergoing combined procedures were more likely to have normal hearing than those having standalone ABR testing (69% vs. 60%; p=0.006). Neurological comorbidities showed no significant association.

Conclusion

Comorbidities, particularly cardiac and respiratory conditions, and procedural context were stronger predictors of ABR outcomes than anesthesia type. These findings suggest anesthetic choice may not independently alter ABR thresholds, emphasizing the importance of considering patient health status and procedural setting when interpreting pediatric ABR results.

## Introduction

Auditory brainstem response (ABR) testing is a diagnostic tool used to assess hearing in pediatric patients, particularly those with suspected hearing loss. This objective measure evaluates the response to sound stimuli and is often performed under general anesthesia in young children who are unable to reliably test while awake [1].

The choice of anesthesia during ABR testing is a crucial consideration, as anesthetic agents have been reported to influence the accuracy and reliability of testing thresholds [2]. Propofol, a commonly used intravenous anesthetic, and inhalation agents such as sevoflurane, are two frequently employed anesthesia types during ABR testing [3]. Both have distinct pharmacological profiles that could theoretically impact the auditory responses measured during testing. While propofol provides rapid induction and recovery times, inhalation agents like sevoflurane may prolong the testing duration but offer ease of administration in certain cases [4]. Controversies exist in the current literature with some studies suggesting decreased reliability of ABR results with the use of sevoflurane, while others demonstrating no significant differences in reliability by anesthetic agent [3-5].

Children undergoing ABR testing often present with various comorbidities, such as respiratory or cardiovascular conditions, which could further complicate anesthesia management and influence ABR outcomes [6]. Additionally, the type of ABR procedure, whether conducted as a standalone procedure or in combination with other interventions, may theoretically impact the duration of anesthesia exposure and, consequently, test results [7]. There is a paucity of current literature regarding the impact of comorbidity and whether ABR is performed in conjunction with other procedures.

This study aimed to evaluate how patient comorbidities and procedural context (standalone versus combined procedures) influence auditory brainstem response (ABR) outcomes in children with suspected hearing loss. In addition, we explored whether the type of anesthetic used (propofol versus sevoflurane) contributed to differences in ABR thresholds. By reframing these questions within a real-world clinical setting, this study sought to clarify previous inconsistencies in the literature regarding anesthetic effects and to highlight the role of underlying medical conditions in ABR interpretation. We hypothesized that comorbidities, particularly cardiac and respiratory conditions, and procedural factors would have a greater impact on ABR outcomes than anesthetic choice. Through this analysis, we aimed to provide evidence to guide clinical decision-making in pediatric otolaryngology, audiology, and anesthesia and to improve the accuracy and interpretability of ABR testing in children with suspected hearing impairments.

## Materials and methods

With approval from the Office for the Protection of Human Subjects and the Institutional Review Board at Children’s National Hospital (IRB Approval #15769), we conducted a retrospective review of an internal dataset of pediatric patients who underwent ABR testing between October 2020 and 2023. A total of 403 patient charts were reviewed, with no exclusion criteria. The demographic variables collected included gender, ethnicity, insurance type, household income, zip code, age, and primary language spoken at home.

In addition to demographic data, the following clinical characteristics were examined: medical comorbidities, specifically those affecting the neurologic, cardiovascular, and respiratory systems; ABR test thresholds (click and 500 and 4000 Hz tone burst frequencies); procedure type (standalone ABR or combined with other procedures); American Society of Anesthesiologists (ASA) class [8]; anesthesia type used during the procedure (sevoflurane, propofol, or both); whether premedication was administered (yes or no); method of anesthesia induction (via endotracheal tube or mask); and the total duration of the procedure and PACU stay. No intraoperative or postoperative complications were recorded for the reviewed cohort.

The relationships between demographic, social, and clinical characteristics of ABR testing were compared using univariate and multivariate analyses to examine the relationships between ABR findings and the type of anesthesia used (sevoflurane vs. sevoflurane combined with or solely propofol) controlling for various clinical and demographic factors, including the presence of comorbidities. Propofol data was not analyzed on its own due to minimal sevoflurane exposure at induction. Hearing loss was categorized into two groups, “normal” and “abnormal”, with abnormal referring to mild, moderate, severe, and profound hearing loss. For the multivariate analyses, adjustments were made based on ASA class to control for patient health status. Odds ratios (ORs) with corresponding 95% confidence intervals (CIs) were calculated to quantify associations, with significance levels assessed through p-values from Chi-square tests or Fisher's exact tests where appropriate. Further analyses included stratification by left and right ear to detect potential side-specific effects. Additional subgroup analyses were performed to evaluate hearing outcomes in patients with specific comorbidities (neurologic, cardiac, and respiratory abnormalities) who had the corresponding diagnostic codes in their problem list and in those undergoing standalone ABR versus combined procedures.

## Results

Patient demographics and clinical characteristics

A total of 403 patients (806 ears) were included in this analysis. The median age of the cohort was 30 months (IQR: 16.0-52.0), with 62.0% (n=250) of the patients being male and 38.0% (n=153) female (Table 1). The racial distribution included 22.6% White (n=91), 32.1% Black or African American (n=129), and 45.3% from other racial groups (n=182). Ethnicity data revealed that 55.2% (n=222) of patients identified as Not Hispanic/Latino, while 26.6% (n=107) were Hispanic/Latino; 18.2% (n=73) did not report their ethnicity. English was the primary language for 69.0% (n=278) of participants, with Spanish spoken by 26.3% (n=106), and other languages by 4.7% (n=19) (Table 1). Income distribution was relatively balanced, with 49.6% (n=200) of patients being from a zip code with a household income below $100,000, and 50.4% (n=203) above $100,000.

**Table 1:** Demographic and clinical characteristics of children with ABRs IQR = interquartile range; ASA = American Society of Anesthesiologists; HL = hearing loss; ABR = auditory brainstem response

Characteristics	Level	Overall
Number of patients (n)		403
Age (months)	Median [IQR]	30.0 [16.0-52.0]
Gender, n (%)	Male	250 (62.0)
	Female	153 (38.0)
Race, n (%)	White	91 (22.6)
	Black or African American	129 (32.1)
	Other	182 (45.3)
Ethnicity, n (%)	Not Hispanic/Latino	222 (55.2)
	Hispanic/Latino	107 (26.6)
	Not reported	73 (18.2)
Language spoken, n (%)	English	278 (69.0)
	Spanish	106 (26.3)
	Other	19 (4.7)
State, n (%)	District of Columbia	53 (13.2)
	Maryland	193 (48.0)
	Virginia	152 (37.8)
	Other	4 (1.0)
Income, n (%)	<100,000	200 (49.6)
	>100,000	203 (50.4)
ASA class, n (%)	1	57 (14.2)
	2	194 (48.3)
	3	149 (37.1)
	4	2 (0.5)
Comorbidities, n (%)	No	268 (67.2)
	Yes	131 (32.8)
Neuro abnormalities, n (%)	No	210 (52.1)
	Yes	193 (47.9)
Cardio abnormalities, n (%)	No	317 (78.7)
	Yes	86 (21.3)
Resp abnormalities, n (%)	No	325 (80.6)
	Yes	78 (19.4)
Intraoperative click (Right), n (%)	None	251 (64.7)
	Mild/Moderate	78 (20.1)
	Severe/Profound	59 (15.2)
HL 500 Hz (Right), n (%)	None	189 (49.6)
	Mild/Moderate	123 (32.3)
	Severe/Profound	69 (18.1)
HL 4000 Hz (Right), n (%)	None	218 (56.0)
	Mild/Moderate	93 (23.9)
	Severe/Profound	78 (20.1)
Intraoperative click (Left), n (%)	None	245 (63.1)
	Mild/Moderate	77 (19.8)
	Severe/Profound	66 (17.0)
HL 500 Hz (Left), n (%)	None	204 (53.8)
	Mild/Moderate	109 (28.8)
	Severe/Profound	66 (17.4)
HL 4000 Hz (Left), n (%)	None	218 (56.0)
	Mild/Moderate	95 (24.4)
	Severe/Profound	76 (19.5)
Standalone ABR, n (%)	No	201 (49.9)
	Yes	202 (50.1)
Procedure, n (%)	ABR	210 (52.2)
	ABR + otology procedure	124 (30.8)
	ABR + non-otology procedure	68 (16.9)
Procedure anesthesia, n (%)	Sevoflurane only	98 (24.4)
	Sevoflurane + Propofol/Only Propofol	303 (75.6)
Procedure time (minutes)	Median [IQR]	40.0 [27.0-55.0]

The ASA classification, which is used to assess a patient’s preoperative physical status and anesthesia risk, was also recorded. Within this cohort, 14.2% (n=57) were classified as ASA 1, 48.3% (n=194) as ASA 2, 37.1% (n=149) as ASA 3, and 0.5% (n=2) as ASA 4 (Table 1). Furthermore, 260 of 403 patients (64.5%) were premedicated.

Regarding medical comorbidities, 32.8% (n=131) of patients had documented conditions, with 47.9% (n=193) presenting with neurological abnormalities, 21.3% (n=86) with cardiac abnormalities, and 19.4% (n=78) with respiratory abnormalities (Table 1). Down syndrome was a notable comorbidity, observed in 72 patients (17.9%).

Anesthesia type and degree of hearing loss

Hearing Loss Distribution Across Anesthesia Types

ABR threshold results across three frequencies (intraoperative click, 500 Hz, and 4000 Hz toneburst) for each ear (right and left), were compared for patients who received only sevoflurane to those who received sevoflurane with propofol or propofol alone. For intraoperative clicks in the right ear, 60.6% (n=57) of the sevoflurane-only group showed no hearing loss, while 66.2% (n=194) of those receiving sevoflurane combined with propofol or propofol alone had no hearing loss (p=0.572). For the left ear, the corresponding percentages were 61.1% (n=58) for the sevoflurane-only group and 64.0% (n=187) for the combination anesthesia group (p=0.869).

At 500 Hz in the right ear, 44.7% (n=42) of patients receiving sevoflurane alone exhibited no hearing loss, compared to 50.9% (n=145) of those receiving combination anesthesia (p=0.420). The left ear showed similar results, with 45.7% (n=43) of the sevoflurane-only group and 56.2% (n=159) of the combination group displaying no hearing loss (p=0.193).

For the 4000 Hz frequency, in the right ear, 52.1% (n=50) of patients in the sevoflurane-only group had no hearing loss, while 57.4% (n=167) in the combination anesthesia group showed no hearing loss (p=0.359). In the left ear at 4000 Hz, 53.6% (n=52) in the sevoflurane-only group had no hearing loss, compared to 56.6% (n=164) in the combination group (p=0.321) (Table 2).

**Table 2:** ABR results by anesthesia approach: sevoflurane vs. sevoflurane + propofol/only propofol * p-values were obtained from Chi-square test/Fisher's exact test; HL = hearing loss

Hearing Loss level	Right side			Left side		
	Procedure			Procedure		
	Sevoflurane	Sevoflurane + propofol/only propofol	P value	Sevoflurane	Sevoflurane + propofol/only propofol	P value
Number of patients (n)	98	303		98	303	
Intraop click, n (%)			0.572			0.869
None	57 (60.6)	194 (66.2)		58 (61.1)	187 (64.0)	
Mild/Moderate	20 (21.3)	57 (19.5)		20 (21.1)	56 (19.2)	
Severe/Profound	17 (18.1)	42 (14.3)		17 (17.9)	49 (16.8)	
HL 500 Hz, n (%)			0.42			0.193
None	42 (44.7)	145 (50.9)		43 (45.7)	159 (56.2)	
Mild/Moderate	31 (33.0)	92 (32.3)		33 (35.1)	76 (26.9)	
Severe/Profound	21 (22.3)	48 (16.8)		18 (19.1)	48 (17.0)	
HL 4000 Hz, n (%)			0.359			0.321
None	50 (52.1)	167 (57.4)		52 (53.6)	164 (56.6)	
Mild/Moderate	28 (29.2)	64 (22.0)		29 (29.9)	66 (22.8)	
Severe/Profound	18 (18.8)	60 (20.6)		16 (16.5)	60 (20.7)	

Logistic Regression Analysis of Hearing Loss and Anesthesia Types

A univariate and multivariate logistic regression analysis was conducted to assess the association between anesthesia type (sevoflurane only vs. sevoflurane combined with propofol or propofol alone) and hearing loss, with adjustments for ASA classification. The analysis categorized hearing outcomes as “no hearing loss” versus “any hearing loss” to simplify interpretation.

In the right ear at the intraoperative click frequency, the odds ratio (OR) for hearing loss in the combination anesthesia group was 0.79 (95% CI: 0.49-1.28, p=0.325) in univariate analysis, and the adjusted odds ratio (aOR) was 0.80 (95% CI: 0.50-1.31, p=0.376) when controlling for ASA classification. At 500 Hz in the right ear, the aOR was 0.79 (95% CI: 0.49-1.27, p=0.339), indicating no significant association between anesthesia type and hearing loss. In the right ear at 4000 Hz, the aOR was 0.84 (95% CI: 0.53-1.34, p=0.468).

In the left ear, similar patterns were observed. For the intraoperative click frequency, the aOR in the combination group was 0.88 (95% CI: 0.55-1.43, p=0.608). At 500 Hz, the aOR was 0.67 (95% CI: 0.41-1.07, p=0.094), and at 4000 Hz, the aOR was 0.89 (95% CI: 0.56-1.43, p=0.633), indicating no significant differences between anesthesia types across these frequencies after adjusting for ASA status (Table 3).

**Table 3:** Univariate and multivariate analysis to explore the relationship of hearing loss and anesthesia type. *adjusted for ASA status; OR = odds ratio; aOR = adjusted odds ratio; CI = confidence interval; Ref = reference group; HL = hearing loss

Outcome	Procedure anesthesia	n (%)		Unadjusted		Adjusted*	
		No HL	HL	OR (95% CI)	P value	aOR (95% CI)	P value
Right side							
Intraoperative click	Sevoflurane	57 (60.6)	37 (39.4)	Ref	Ref	Ref	Ref
	Sevoflurane + propofol/only propofol	194 (66.2)	99 (33.8)	0.79 (0.49-1.28)	0.325	0.80 (0.50-1.31)	0.376
HL 500 Hz	Sevoflurane	42 (44.7)	52 (55.3)	Ref	Ref	Ref	Ref
	Sevoflurane + propofol/only propofol	145 (50.9)	140 (49.1)	0.78 (0.49-1.24)	0.298	0.79 (0.49-1.27)	0.339
HL 4000 Hz	Sevoflurane	50 (52.1)	46 (47.9)	Ref	Ref	Ref	Ref
	Sevoflurane + propofol/only propofol	167 (57.4)	124 (42.6)	0.81 (0.51-1.28)	0.364	0.84 (0.53-1.34)	0.468
Left side							
Intraop click	Sevoflurane	58 (61.1)	37 (38.9)	Ref	Ref	Ref	Ref
	Sevoflurane + propofol/only propofol	187 (64.0)	105 (36.0)	0.88 (0.55-1.42)	0.600	0.88 (0.55-1.43)	0.608
HL 500 Hz	Sevoflurane	43 (45.7)	51 (54.3)	Ref	Ref	Ref	Ref
	Sevoflurane + propofol/only propofol	159 (56.2)	124 (43.8)	0.66 (0.41-1.05)	0.080	0.67 (0.41-1.07)	0.094
HL 4000 Hz	Sevoflurane	52 (53.6)	45 (46.4)	Ref	Ref	Ref	Ref
	Sevoflurane + propofol/only propofol	164 (56.6)	126 (43.4)	0.89 (0.56-1.41)	0.613	0.89 (0.56-1.43)	0.633

Hearing loss and patient comorbidities

Seventy-eight (19.4%) patients from the cohort had documented respiratory comorbidities. These conditions included bronchopulmonary dysplasia (BPD; n=7, 1.7%), chronic lung disease (n=7, 1.7%), asthma (n=20, 5.0%), pulmonary stenosis (n=1, 0.2%), recurrent pneumonia infections (n=5, 1.2%), sleep-disordered breathing (n=4, 1.0%), sleep apnea (n=6, 1.5%), diaphragm paralysis (n=1, 0.2%), obstructive sleep apnea (OSA; n=10, 2.5%), stridor (n=6, 1.5%), reactive airway disease (n=2, 0.5%), subglottic stenosis (SGS; n=1, 0.2%), pulmonary hypertension (HTN; n=1, 0.2%), and upper respiratory infections (URI; n=7, 1.7%). All patients with BPD (n=7, 1.7%) were born prematurely. Gestational ages were as follows: 23 weeks, 24 weeks, 25 weeks, 26 weeks, 27 weeks, and 28 weeks. Respiratory support included intubation (n=5, 1.2%) and tracheostomy and ventilator dependency (n=2, 0.5%). Significant associations in ABR results were observed in patients with chronic respiratory co-morbidities, particularly at 500 Hz and 4000 Hz. For example, in the left ear at 500 Hz, 56.9% (n=41) of patients without respiratory issues had no hearing loss, compared to 39.7% (n=27) of those with respiratory abnormalities (p=0.013). At 4000 Hz in the right ear, 57.3% (n=181) of patients without respiratory issues exhibited no hearing loss, compared to 50.7% (n=37) of those with respiratory issues (p=0.024) (Table 4).

**Table 4:** Hearing loss level in patients with respiratory comorbidities * p-values obtained from Chi-square test/Fisher's exact test; HL = hearing loss

Hearing Loss level	Right side			Left side		
	Resp. abnormalities			Resp. abnormalities		
	No	Yes	P value	No	Yes	P value
Number of patients (n)	325	78		325	78	
Intraoperative click, n (%)			0.117			0.035
None	205 (65.1)	46 (63.0)		204 (64.6)	41 (56.9)	
Mild/Moderate	58 (18.4)	20 (27.4)		55 (17.4)	22 (30.6)	
Severe/Profound	52 (16.5)	7 (9.6)		57 (18.0)	9 (12.5)	
HL 500 Hz, n (%)			0.305			0.013
None	161 (51.4)	28 (41.2)		177 (56.9)	27 (39.7)	
Mild/Moderate	97 (31.0)	26 (38.2)		80 (25.7)	29 (42.6)	
Severe/Profound	55 (17.6)	14 (20.6)		54 (17.4)	12 (17.6)	
HL 4000 Hz, n (%)			0.024			0.014
None	181 (57.3)	37 (50.7)		183 (57.7)	35 (48.6)	
Mild/Moderate	67 (21.2)	26 (35.6)		68 (21.5)	27 (37.5)	
Severe/Profound	68 (21.5)	10 (13.7)		66 (20.8)	10 (13.9)	

Eighty-six patients (21.3%) from the cohort had documented cardiac comorbidities. The specific cardiac conditions and the corresponding number of patients affected were as follows: ventricular septal defect (VSD; n=14, 3.5%), atrioventricular canal defect (AV Canal Defect; n=5, 1.2%), patent ductus arteriosus (PDA; n=6, 1.5%), patent foramen ovale (PFO; n=4, 1.0%), heart murmur (n=18, 4.5%), atrioventricular septal defect (AVSD; n=12, 3.0%), supraventricular tachycardia (SVT; n=1, 0.2%), hemophilia (n=1, 0.2%), and congenital heart disease (CHD; n=25, 6.2%). Patients with cardiac comorbidities showed significant differences in hearing loss at 500 Hz in the right ear. Specifically, 53.2% (n=160) of patients without cardiac abnormalities had no hearing loss, while only 36.2% (n=29) of those with cardiac conditions were in this category (p=0.008). No significant differences were found at 4000 Hz or in the left ear (Table 5).

**Table 5:** Hearing loss level in patients with cardiac comorbidities * p-values were obtained from Chi-square test/Fisher's exact test; HL = hearing loss

Hearing Loss level	Right side			Left side		
	Cardiac abnormalities			Cardiac abnormalities		
	No	Yes	P value	No	Yes	P value
Number of patients (n)	317	86		317	86	
Intraoperative click, n (%)			0.564			0.156
None	195 (64.4)	56 (65.9)		195 (64.4)	50 (58.8)	
Mild/Moderate	59 (19.5)	19 (22.4)		54 (17.8)	23 (27.1)	
Severe/Profound	49 (16.2)	10 (11.8)		54 (17.8)	12 (14.1)	
HL 500 Hz, n (%)			0.008			0.052
None	160 (53.2)	29 (36.2)		166 (55.7)	38 (46.9)	
Mild/Moderate	86 (28.6)	37 (46.2)		77 (25.8)	32 (39.5)	
Severe/Profound	55 (18.3)	14 (17.5)		55 (18.5)	11 (13.6)	
HL 4000 Hz, n (%)			0.105			0.172
None	171 (55.5)	47 (58.0)		173 (56.5)	45 (54.2)	
Mild/Moderate	69 (22.4)	24 (29.6)		69 (22.5)	26 (31.3)	
Severe/Profound	68 (22.1)	10 (12.3)		64 (20.9)	12 (14.5)	

Among patients with neurological comorbidities, there were no significant differences in hearing loss at any frequency in either ear (Table 6). For instance, at 500 Hz in the right ear, 47.3% (n=95) of patients without neurological abnormalities had no hearing loss, compared to 52.2% (n=94) of those with neurological abnormalities (p=0.293).

**Table 6:** Hearing loss level in patients with neurological comorbidities * p-values were obtained from Chi-square test/Fisher's exact test; HL = hearing loss

Hearing Loss level	Right side			Left side		
	Neuro abnormalities			Neuro abnormalities		
	No	Yes	P value	No	Yes	P value
Number of patients (n)	210	193		210	193	
Intraop click, n (%)			0.884			0.985
None	129 (63.5)	122 (65.9)		128 (62.7)	117 (63.6)	
Mild/Moderate	42 (20.7)	36 (19.5)		41 (20.1)	36 (19.6)	
Severe/Profound	32 (15.8)	27 (14.6)		35 (17.2)	31 (16.8)	
HL 500 Hz, n (%)			0.293			0.745
None	95 (47.3)	94 (52.2)		107 (53.0)	97 (54.8)	
Mild/Moderate	72 (35.8)	51 (28.3)		57 (28.2)	52 (29.4)	
Severe/Profound	34 (16.9)	35 (19.4)		38 (18.8)	28 (15.8)	
HL 4000 Hz, n (%)			0.676			0.21
None	110 (54.2)	108 (58.1)		111 (53.6)	107 (58.8)	
Mild/Moderate	52 (25.6)	41 (22.0)		58 (28.0)	37 (20.3)	
Severe/Profound	41 (20.2)	37 (19.9)		38 (18.4)	38 (20.9)	

Impact of standalone versus combined ABR procedures on hearing loss levels

Differences in hearing loss were observed between standalone ABR procedures and those combined with other interventions. For intraoperative click measurements in the right ear, 69.4% (n=134) of patients who underwent combined procedures had no hearing loss compared to 60.0% (n=117) in the standalone ABR group (p=0.006). In the left ear, combined procedures similarly showed a higher percentage of no hearing loss (n=133, 68.9%) compared to standalone ABR (n=112, 57.4%) (p=0.010). At 4000 Hz in the right ear, 59.2% (n=113) of patients in the combined procedure group had no hearing loss, compared to 53.0% (n=105) in the standalone group (p=0.006) (Table 7).

**Table 7:** Hearing levels due to stand-alone ABR vs. combined procedure * p-values were obtained from Chi-square test/Fisher's exact test; HL = hearing loss; ABR = auditory brainstem testing

Hearing Loss level	Right side			Left side		
	Procedure			Procedure		
	Combined procedure	Stand-alone ABR	P value	Combined procedure	Stand-alone ABR	P value
Number of patients (n)	201	202		201	202	
Intraoperative click, n (%)			0.006			0.01
None	134 (69.4)	117 (60.0)		133 (68.9)	112 (57.4)	
Mild/Moderate	41 (21.2)	37 (19.0)		38 (19.7)	39 (20.0)	
Severe/Profound	18 (9.3)	41 (21.0)		22 (11.4)	44 (22.6)	
HL 500 Hz, n (%)			0.206			0.005
None	89 (48.6)	100 (50.5)		100 (54.6)	104 (53.1)	
Mild/Moderate	66 (36.1)	57 (28.8)		62 (33.9)	47 (24.0)	
Severe/Profound	28 (15.3)	41 (20.7)		21 (11.5)	45 (23.0)	
HL 4000 Hz, n (%)			0.006			0.004
None	113 (59.2)	105 (53.0)		113 (58.9)	105 (53.3)	
Mild/Moderate	52 (27.2)	41 (20.7)		54 (28.1)	41 (20.8)	
Severe/Profound	26 (13.6)	52 (26.3)		25 (13.0)	51 (25.9)	

## Discussion

This study showed no evidence of differences in hearing threshold results based on auditory brainstem response (ABR) testing between patients who received either sevoflurane alone and a combined anesthetic approach (sevoflurane and propofol) or propofol alone. However, significant differences were observed in the presence of hearing loss in patients with cardiac and respiratory comorbidities compared to those without these conditions; patients with these comorbidities were less likely to have no hearing loss. Additionally, patients undergoing combined surgical procedures were less likely to display hearing loss than those undergoing ABR testing only.

Our findings provide a unique perspective on the impact of anesthetic type, procedural context, and patient comorbidities on ABR outcomes. While previous research suggested that propofol might produce fewer false positives for hearing loss compared to sevoflurane, our study did not detect significant differences in hearing loss rates between patients receiving inhalational anesthetic alone and those receiving a combined approach with propofol [3]. This may indicate that, in our patient population, anesthesia choice does not substantially influence ABR positivity rate while other clinical factors are more likely to be associated.

Research on pediatric ABR testing has primarily focused on the safety and complication rates of various sedation protocols, with limited emphasis on how these protocols may independently impact hearing outcomes in patients with comorbidities [6]. Our study addresses this underrecognized gap by examining the influence of cardiac and respiratory conditions on hearing outcomes, showing that patients with these comorbidities are more likely to exhibit hearing loss. This highlights the importance of accounting for specific health factors in ABR interpretation, as underlying medical conditions may be associated with the likelihood of detecting hearing loss.

In particular, our findings demonstrated that children with cardiac comorbidities were significantly more likely to exhibit hearing loss at 500 Hz in the right ear compared to those without cardiac abnormalities, with our data for the left ear close to significance. These results align with prior studies identifying CHD as a risk factor for sensorineural hearing loss (SNHL) due to shared embryological and pathological mechanisms. Neurodevelopmental injuries in children with CHD are believed to stem from embryonic circulatory impairments, as well as hemodynamic and metabolic stressors during the neonatal period, particularly those associated with complex cardiac surgical interventions [9]. Perioperative factors such as prolonged deep hypothermic circulatory arrest, low arterial oxygen tension, and the use of ototoxic medications like furosemide and high-dose aspirin have been implicated in the development of SNHL in this population [9-11].

The prevalence of SNHL in children undergoing early cardiac surgery is reported to range from 3.9% to 6.9%, highlighting the significant impact of CHD and its treatment on auditory outcomes. The co-occurrence of CHD and SNHL may also arise from genetic and developmental dysregulation [12-14]. Syndromes such as CHARGE, Turner Syndrome, 22q11.2 deletion, and Noonan syndrome are commonly associated with both conditions, while non-genetic etiologies, such as congenital viral infections, e.g., cytomegalovirus (CMV), rubella, teratogen exposure, and neurodevelopmental insults related to surgical interventions, further contribute to the risk [12-15].

Universal newborn screening for hearing loss and critical CHD has improved the early identification of these conditions, providing opportunities for timely intervention. Notably, studies have reported cardiac abnormalities in 5-6% of children undergoing cochlear implantation, with early cardiac surgery and CMV infection being the most frequent non-genetic etiologies [16]. Genetic analyses have revealed CHARGE syndrome as the most common cause of co-occurring CHD and SNHL, followed by other syndromes such as Turner syndrome, 22q11.2 deletion, and Noonan syndrome [15, 17].

Similarly, our findings demonstrated that children with respiratory comorbidities were significantly more likely to exhibit hearing loss compared to those without these conditions. Chronic respiratory conditions, such as chronic lung disease (CLD), often result in periods of chronic or intermittent hypoxia, which can adversely impact the auditory system. Studies have shown that children with CLD demonstrate delayed auditory P300 latencies and prolonged ABR wave latencies, suggesting retrocochlear involvement and an increased incidence of SNHL [18]. Hypoxia during critical stages of development is thought to impair the central auditory pathways, potentially leading to structural and functional deficits. Additionally, systemic inflammation and metabolic stress associated with chronic respiratory diseases may further exacerbate damage to the auditory system.

These findings underscore the necessity of integrating comprehensive audiological assessments into the care of children with cardiac and respiratory comorbidities. Proactive identification and management of hearing loss, along with minimizing perioperative risks such as hypoxia and ototoxic exposure, may improve outcomes and reduce the burden of SNHL in this population.

Additionally, previous studies have raised concerns about the accuracy of ABR testing conducted in the operating room, especially when performed in conjunction with otologic surgeries such as tympanostomy tubes, as procedural factors may alter auditory responses [7]. Our data furthers this observation by showing that patients who undergo combined surgical procedures, including non-otologic interventions, are less likely to display hearing loss compared to those having standalone ABR testing, suggesting the otolaryngologic surgical intervention often resolves conductive factors potentially affecting hearing outcomes.

This study has several limitations that may affect the generalizability and interpretation of the findings. The retrospective design limited control over potential confounders, including variations in anesthesia administration and patient management. ABR testing was conducted in a clinical operating room setting, which may introduce environmental or procedural influences on auditory responses, as noted in prior literature. Although analyses adjusted for certain demographic and clinical factors, unmeasured variables - such as the specific underlying conditions within cardiac and respiratory comorbidities - may have influenced outcomes. The study population was drawn from a single institution and a specific pediatric cohort, which may limit broader applicability. In addition, selection bias is possible because participants were already undergoing anesthesia, and no a priori power analysis was performed to guide sample size determination.

Overall, this study found no significant differences in the identification of hearing loss through ABR testing based on anesthesia type (inhalation alone vs. combined with propofol). However, the presence of cardiac and respiratory comorbidities was associated with an increased likelihood of hearing loss, highlighting the importance of considering patient health status in interpreting ABR results. Patients who underwent combined surgical procedures exhibited lower rates of hearing loss compared to those undergoing standalone ABR, suggesting that procedural context may play a role in auditory assessment outcomes. These findings underscore the need for clinicians to account for both procedural and patient-specific factors when conducting and interpreting ABR tests in pediatric patients. The absence of significant differences between anesthesia types suggests that the auditory brainstem’s response is relatively robust to the anesthetic agents used in this study, although further research is needed to confirm this in different clinical contexts.

## Conclusions

Our study contributes to a better understanding of how anesthesia type, comorbidities, and procedural context influence ABR outcomes in pediatric patients. Future research should aim to explore these factors in a prospective, multicenter study to better elucidate the complex interactions between anesthetic agents, patient health conditions, and procedural environments in auditory assessments. By expanding our knowledge in this area, we can enhance the accuracy and clinical utility of ABR testing in pediatric audiology.
